# Behaviours indicating cannibalistic necrophagy in ants are modulated by the perception of pathogen infection level

**DOI:** 10.1038/s41598-020-74870-8

**Published:** 2020-10-21

**Authors:** István Maák, Eszter Tóth, Magdalena Lenda, Gábor Lőrinczi, Anett Kiss, Orsolya Juhász, Wojciech Czechowski, Attila Torma

**Affiliations:** 1 Museum and Institute of Zoology, Polish Academy of Sciences, Wilcza 64, 00-679 Warsaw, Poland; 2 Department of Microbiology, University of Szeged, Közép Fasor 52, Szeged, 6726 Hungary; 3 Fungal Pathogenicity Mechanisms Research Group, Hungarian Academy of Sciences, University of Szeged, Közép Fasor 52, Szeged, 6726 Hungary; 4 Australian Research Council Centre of Excellence for Environmental Decisions, School of Biological Sciences, University of Queensland, St. Lucia, QLD 4072 Australia; 5 Institute of Nature Conservation, Polish Academy of Sciences, Mickiewicza 33, 31-120 Kraków, Poland; 6 Department of Ecology, University of Szeged, Közép Fasor 52, Szeged, 6726 Hungary; 7 Doctoral School in Biology, Faculty of Science and Informatics, University of Szeged, Közép Fasor 52, Szeged, 6726 Hungary; 8 Center for Ecological Research, Institute of Ecology and Botany, ʽLendület’ Landscape and Conservation Ecology, Alkotmány Utca 2-4, Vácrátót, 2163 Hungary

**Keywords:** Behavioural ecology, Ecological epidemiology

## Abstract

Cannibalistic necrophagy is rarely observed in social hymenopterans, although a lack of food could easily favour such behaviour. One of the main supposed reasons for the rarity of necrophagy is that eating of nestmate corpses carries the risk of rapid spread of pathogens or parasites. Here we present an experimental laboratory study on behaviour indicating consumption of nestmate corpses in the ant *Formica polyctena*. We examined whether starvation and the fungal infection level of the corpses affects the occurrence of cannibalistic necrophagy. Our results showed that the ants distinguished between corpses of different types and with different levels of infection risk, adjusting their behaviour accordingly. The frequency of behaviours indicating cannibalistic necrophagy increased during starvation, although these behaviours seem to be fairly common in *F. polyctena* even in the presence of other food sources*.* The occurrence and significance of cannibalistic necrophagy deserve further research because, in addition to providing additional food, it may be part of the hygienic behaviour repertoire. The ability to detect infections and handle pathogens are important behavioural adaptations for social insects, crucial for the fitness of both individual workers and the entire colony.

## Introduction

Cannibalism is a taxonomically widespread phenomenon in the animal kingdom but is uncommon in most species^1–4^. It can be divided into two distinct behaviours: killing and eating individuals of the same species or genera (cannibalistic predation), and eating already-dead taxonomically (and even genetically) related individuals (cannibalistic necrophagy); both behaviours have been described in humans^5^. We suggest this distinction for cannibalism in social species, as the terminology is not clarified in the literature^6^. The ability to prevent cannibalistic predation, especially of kin, is particularly important in social groups^7–9^, as it can favour tolerance and kin recognition, that may lead to the evolution of cooperation and all forms of altruism^9^. As a result, in social insects, in most cases, cannibalism takes the form of necrophagy^6^.

Cannibalism might usually be advantageous for the cannibalistic individual by providing essential nutrients, reducing competition and starvation and regulating population densities^2–4,10^. Although social insects often eat their own eggs, larvae and pupae, cannibalism against corpses of adults is considered quite rare in ants. It has not yet been described in wasps and bees, but has been documented in many termite species^6,11–14^. Nevertheless, the role of nestmate corpses as a possible food source is not clear among social insects, as some species manifest conspecific corpse avoidance^6,13^, while others practice cannibalism of closely related individuals (even nestmates) mainly when other food supplies are scarce^6,15,16^. Usually, corpse avoidance is mediated by the appearance of fatty acids on a dead body. These chemicals, associated with death, seem to be phylogenetically ancient signals indicating the threat of being killed posed by e.g. a predator or a disease^13,17^. In many social insects, these compounds induce necrophoresis (the removal of corpses), as one of the most important social prophylactic mechanisms^18–22^. Moreover, the presence of these chemicals or other alterations in the overall cuticular hydrocarbon patterns can also signal internal infection^23^ and induce the killing of pupae^24^, as well as triggering a more aggressive behaviour towards fungus-infected adults in some ants^25^ or cannibalism of moribund adult termites^14^.

Cannibalism can contribute to the control of infestations and diseases by the consumption of infected/parasitized individuals^4,10,14^, although cannibals may also acquire pathogens and spread a disease across the colony^2,26^. However, in a modelling analysis, it was found that the spread of diseases through cannibalism is rare, except in social species with collective corpse consumption^27^. A more general epidemiological model states that the prevalence of vertically transmitted parasites is enhanced by cannibalism when it is not the only mechanism regulating population density^28^. In size-structured populations, both cannibalism and competition seem to be present^29^, so it seems, based on previous models, that cannibalism can have a prominent role in the spread of infectious diseases across a wide range of parasite-host systems^28^. The relative rarity of cannibalism could be explained by the risk of such disease spread-related fitness costs^2,6,27,28,30^. It has been experimentally shown that a single fungal-infected cadaver can be fatal to a whole ant sub-colony of around 20 ants^31^. However, even if handling infected individuals could increase the risk of horizontal transmission, low-level infections acquired through contact with an infected nestmate may even boost the individual immunity system^32–35^.

Although cannibalistic necrophagy of nestmates in many ant species could be an adaptative strategy (just like in termites), detailed studies of this behaviour and descriptions of how ants manage the risk of being infected are still missing. In our study, we aimed to examine experimentally the modulating effect of pathogen contamination and starvation on cannibalistic necrophagy behaviour among nestmates. We chose *Formica polyctena*, as this species is already known to be cannibalistic^15,16,36,37^ and the need for protein can be continuously present in a huge colony containing several (even thousands) queens^38,39^. Although cannibalistic necrophagy can ensure the survival of workers in conditions of food shortage^37^, the characteristics and drivers of corpse necrophagy were not investigated experimentally. In this study, we hypothesized that corpse necrophagy is an important phenomenon present during the handling of nestmate corpses in *F. polyctena*. More specifically we hypothesized that (1) the origin of the corpse (i.e. corpses of potential prey, allospecific corpses, or conspecific corpses) and their state (i.e. old or fresh corpses) can alter the responses of workers and the rate of necrophagy and that (2) conspecific necrophagy is mediated by the nutritional state of the colony (i.e. before, during and after starvation). Based on these, we predicted that (a) ant workers can discriminate between the corpses of potential prey and those of conspecific workers, and there would be a difference in the presence and magnitude of corpse consumption rate between allospecific corpses and those of old and fresh conspecific nestmate corpses. Moreover, (b) the corpse consumption rate would be different before, during and after an experimentally induced food shortage period. Whether corpses were indeed consumed or not is a crucial question in the present study, therefore we aimed to gain further evidence for corpse consumption and discussed the literature in the line with our findings.

Corpse necrophagy might also involve the risk of disease spread-related fitness costs, although this has not investigated experimentally in social insects. As an infection might be a serious threat for social insects, we hypothesised that (3) species exhibiting conspecific necrophagy have efficient pathogen recognition/avoidance mechanisms that mediate conspecific necrophagy. To test this hypothesis, we used the generalist fungal entomopathogen *Beauveria bassiana*, which is a natural pathogen of a wide range of insects including ants of the genus *Formica*^40,41^. *Beauveria bassiana* produces conidia (asexual spores) that can easily attach to the ant cuticle, where these germinate, and the hyphae penetrate through the cuticle into the body and develop in the haemocoel, killing the host and producing a large number of external conidiophores^42^. Based on this hypothesis, we predicted that (c) the handling of corpses infected with fungi and necrophagy will vary depending on the stage of development of the fungus (i.e. spores, hyphae, hyphae with conidia)*.*

## Results

### Responses to the corpses of potential prey, nestmates, and conspecific non-nestmates

Ten freeze killed corpses of potential prey (fruit flies *Drosophila melanogaster*), nestmates, and conspecific non-nestmates were presented to each experimental colony. The corpses were placed on plastic plates inside the foraging arena and we observed the responses of the colonies by noting down the number of corpses and the number of *F. polyctena* workers on the plates, and the behavioural responses of workers towards the different types of corpses.

**Figure 1:**
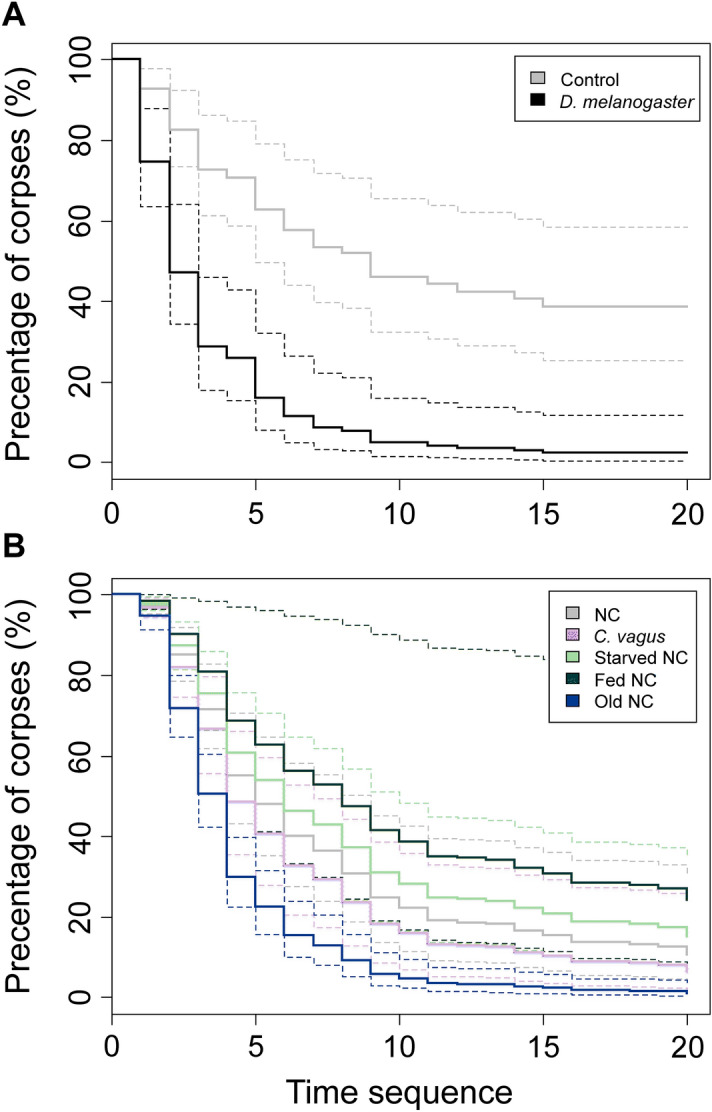
Cox regression of time to corpse removal rate in *Formica polyctena* based on the different type of corpses used during the experimental setups: (**A**) Responses to the corpses of potential prey (*Drosophila melanogaster*) and nestmates (Control); (**B**) Nestmate corpse consumption: *C. vagus*—outer group represented by the corpses of *Camponotus vagus*; NC—nestmate corpses; starved NC—nestmate corpses given to starving colonies; fed NC—nestmate corpses given to satiated colonies; old NC—seven-day-old corpses. Broken lines represent a pointwise 95% confidence interval around the corresponding functions.

The corpses of potential prey were retrieved with the fastest rate (Cox regression *coefficient* ≥ 2.36, *z* ≥ 7.38, *N* = 120, *P* < 0.001; Fig. 1A) and highest amount compared to nestmate and conspecific non-nestmate corpses (GLMM *z* ≥ 3.99, *N* = 120, *P* < 0.001). Moreover, their presence elicited the highest number of workers around the corpses (GLMM *z* ≥ 6.96, *N* = 180, *P* < 0.001) and also a high number of adverse behaviours (*z* = 3.82, *P* < 0.001). This was, however, not significantly different from the number elicited by the non-nestmate corpses (*z* = 0.73, *NS*). On the other hand, non-nestmate corpses elicited a higher number of adverse reactions (*z* = 2.82, *P* < 0.01), but slightly lower activity (*z* = –1.96, *P* = 0.05) than the corpses of nestmates. A higher amount of non-nestmate corpses was retrieved from the plates (*z* = 2.03, *P* < 0.05), but the transport rate (*coefficient* = 0.43, *z* = 1.16, *NS*) did not differ significantly from those of nestmate corpses. The best model also contained the number of individuals and the number of corpses, but neither of them was significant (*z* ≥ 0.94, *NS*).

Comparing the responses to the nestmate corpses used during the different experimental setups, we found no significant difference in the number of adverse reaction or in the number of workers that appeared around the corpses. The best model contained only the number of workers (GLMM *z* = 0.82, *N* = 248, *NS*) and the number of corpses (*z* = 2.8, *P* < 0.01). The corpses were removed in the same amount (GLMM 0.73 ≤ *z* ≤ 1.75, *N* = 180, *NS*) and with the same rate (0.34 ≤ *coefficient* ≤ 1.09, 0.65 ≤ *z* ≤ 1.68, *NS*) during the experiments, so our observations were consistent.

### Corpse retrieval and cannibalistic necrophagy altered by starvation

This experiment was performed with ant corpses of *C. vagus* as an outgroup, followed by experiments with nestmate corpses (NC hereafter) with colonies under normal feeding regime (control), subjected to a seven-day starvation period (starved colonies hereafter), and also one week after the normal feeding regime (non-starved colonies hereafter) was retaken. The last experiment was performed by using seven-day-old nestmate corpses (old NC hereafter). The corpses were considered consumed, if the weight loss of corpses before the experiment and after they were brought out from the nest was two times larger than the mean weight loss of the controls.

In each setup, the majority of corpses were carried away from the plates to the nests (Table 1). Old NC were retrieved from the plates at a faster rate than any other corpse type (Cox regression *coefficient* ≥ 0.65, *z* ≥ 3.32, *P* ≤ 0.002; Fig. 1B). The second most promptly retrieved corpses were those of *C. vagus*, which were transported at a faster rate than NC in starved and non-starved colonies (*coefficient* ≥ 0.57, *z* ≥ 2.85, *P* ≤ 0.007; Fig. 1B). On the other hand, a greater number of workers appeared around the NC in starved colonies as compared to the corpses of *C. vagus* (GLMM *z* = 3.01, *N* = 348, *P* = 0.005), while the fewest individuals were observed around the NC in non-starved colonies (*z* ≥ 3.46, *P* < 0.001). The workers from starved colonies started to consume the corpses on the plates and dragged them inside the nests only with a delay. Overall, significantly lower numbers of NC were transported away from the plates in non-starved colonies (every other corpse type: GLMM *z* ≤ –3.18, *N* = 300, *P* < 0.003). However, one hour after the end of the experiment, the majority of the corpses were transported to the nest, except the old NC (every other corpse type: GLMM *z* ≤ –3.1, *N* = 300, *P* < 0.004) (Table 1). The majority of old NC were still dragged in different directions in the arena, but after 12 h, all corpses were transported to the nests. The other differences regarding the transport rate (0.06 ≤ coefficient ≤ 0.38, 0.29 ≤ *z* ≤ 1.66, *NS*), the number of workers present around the corpses (0.56 ≤ z ≤ 1.84, *NS*) and the effect of the number of corpses (z = 0.69, *NS*) were not significant.

**Table 1:** The percentage of corpses removed from the plates during the observation period and taken to the nests 1, 3 and 12 h after the observation period in the different setups of corpse consumption analysis of *F. polyctena*. The last column shows the number of corpses found on the cemetery after they were firstly brought into the nest.

Corpse type	Removed from plates (within 1 h)	Transported into the nest after 1 h	Transported into the nest after 3 h	Transported into the nest after 12 h	Transported to the cemetery from the nest
Corpses of CC	90	82	83	100	85
Corpses of NC	90	80	80	100	92
Corpses of starved NC	95	87	95	100	92
Corpses of fed NC	68	75	93	100	78
Corpses of old NC	100	53	72	100	80

In the different columns, the percentages of the total number of corpses are shown. CC—outgroup represented by the corpses of *Camponotus vagus*; NC—nestmate corpses; starved NC—nestmate corpses given to starving colonies; fed NC—nestmate corpses given to satiated colonies; old NC—seven-day-old corpses.

### Corpse disposal and cannibalistic necrophagy altered by starvation

**Figure 2:**
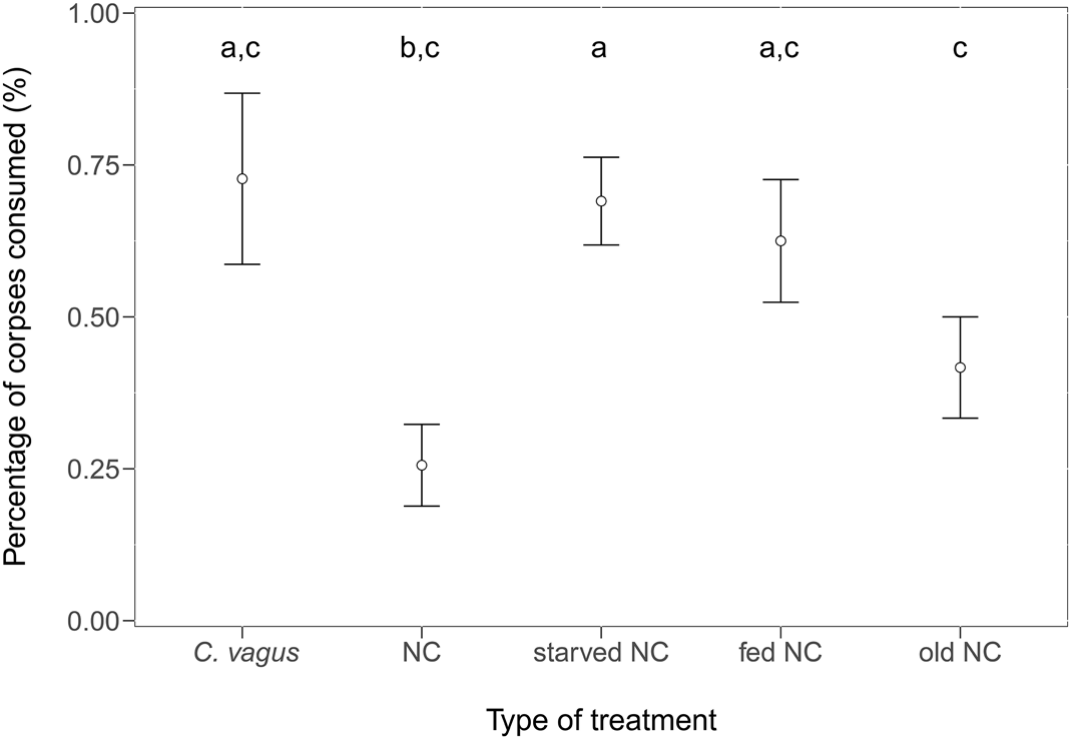
Percentage (% ± SE) of different corpses consumed in *Formica polyctena* in different treatment types: *C. vagus*—outgroup represented by the corpses of *Camponotus vagus*; NC—nestmate corpses; starved NC—nestmate corpses given to starving colonies; fed NC—nestmate corpses given to satiated colonies; old NC—seven-day-old corpses. Different letters above boxes represent groups that differ significantly from each other.

The majority of corpses were brought outside from the nest one to five days after the start of the experiment. The corpses of *C. vagus* (Cox regression *coefficient* ≥ 0.51, *z* ≥ 2.49, *N* = 300, *P* < 0.05) and NC in non-starved colonies (*coefficient* ≥ 0.5, *z* ≥ 2.36, *P* < 0.05) were retrieved significantly more slowly than any other corpse type. There were no other significant differences among the other corpse types in this respect (0.001 ≤ *coefficient* ≤ 0.3, 0.01 ≤ *z* ≤ 1.59, *NS*). The corpses of *C. vagus* were consumed in the highest amounts; this result was significantly different from that of the control NC (GLMM *z* = 2.9, *N* = 156, *P* < 0.05; Fig. 2). Moreover, the corpses of *C. vagus* were also more frequently brought out in pieces (separate head, mesosoma, gaster) than the control NC (GLMM *z ≤ *–3.66, *N* = 256, *P* < 0.001). Nestmate corpses were consumed in the greatest percentage in starved colonies, which was significantly different from the percentages for the control and old NC (41.67%, *z* ≥ 2.55, *P* < 0.05; Fig. 2). Surprisingly, the NC in non-starved colonies were also consumed in a greater percentage than in the control colonies (*z* = 3.34, *P* < 0.01; Fig. 2), and in the non-starved colonies more NC were brought out in pieces than every other type of NC (*z* > 2.15, *P* < 0.05). All other differences were not significant (0.3 ≤ z ≤ 1.61, *NS*).

### Cannibalistic necrophagy and pathogen recognition

This experiment was performed with nestmate corpses without infection (controls) and infected with different developmental stages of the generalist fungal entomopathogen *B. bassiana* (spore-contaminated corpses, corpses with hyphae and sporulating corpses).

**Figure 3:**
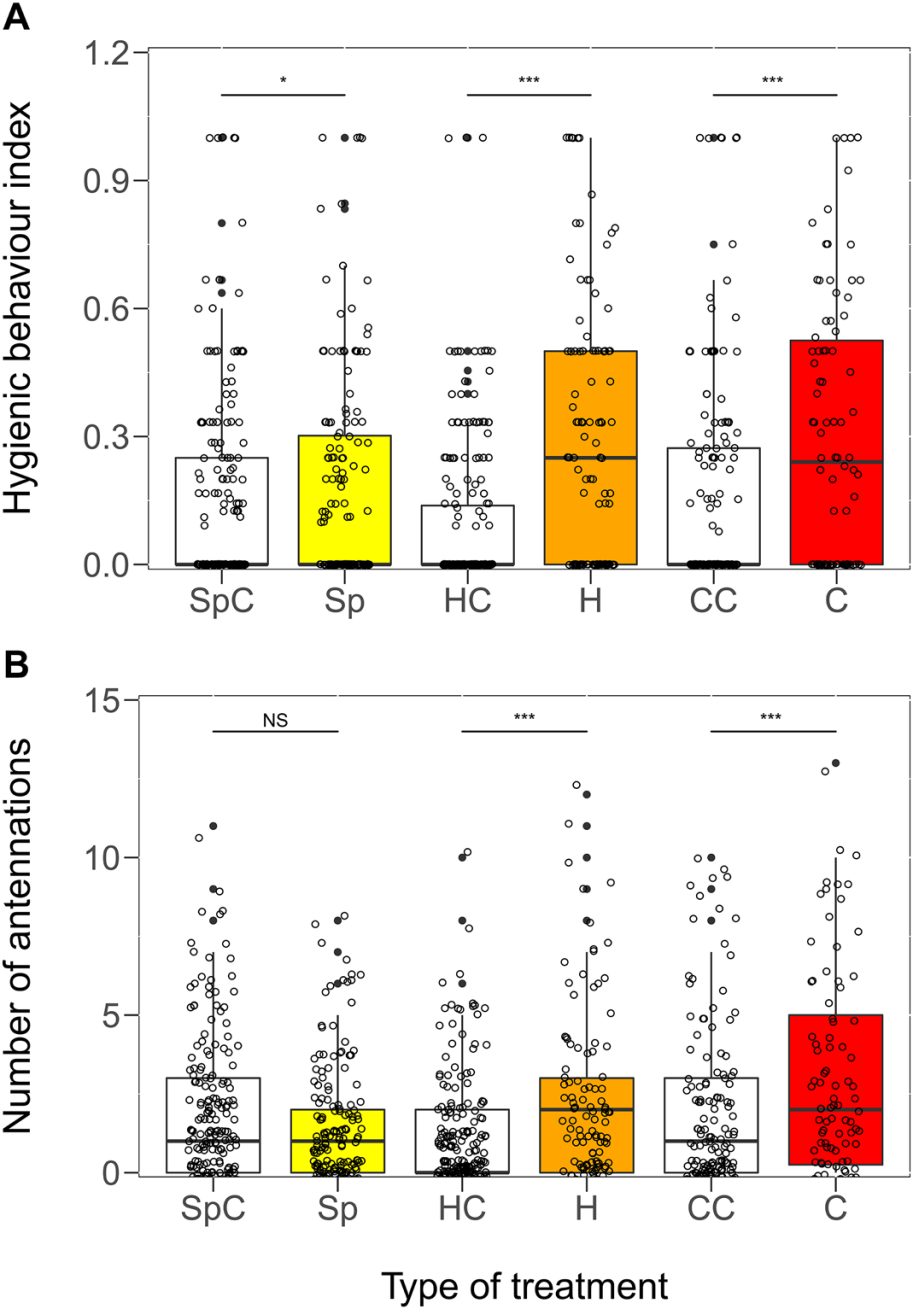
Hygienic behaviours (**A**) and the number of antennations (**B**) recorded in *F. polyctena* in the presence of control corpses (white) and corpses infected with spores (yellow), hyphae (orange) and hyphae with conidia (red) of *Beauveria bassiana* (medians, quartiles and range; *N*_*1-min observations*_ = 887). SpC—spore control; Sp—contaminated with spores; HC—hyphae control; H—infected with hyphae; CC—sporulating corpse control; C—sporulating corpses. Box plots show median, quartiles, min–max values, outliers (black dots) and individual data points (empty circles). Different marks above boxes represent the different levels of significance. (*NS*—non-significant, *P < 0.05, ***P < 0.001).

The corpses with hyphae and those that were sporulating elicited more hygienic behaviours and antennations than those of the controls and the corpses contaminated with spores, while there was no significant difference between the two former treatment types (Table 2; Fig. 3A,B). The sporulating corpses were transported away from the plates in higher amounts and at a higher rate than those contaminated with spores and hyphae (Table 2). The sporulating corpses were never observed to be transported to the nest and were groomed even after 24 h in the cemeteries. The corpses treated with hyphae were transported in higher amounts and at a higher rate than those contaminated with spores (Table 2; Fig. 3A,B), and some of the corpses with hyphae were transported to the nests. The corpses contaminated with spores elicited more hygienic behaviours than the controls did, but the number of antennations was similar (Table 2; Fig. 3A,B). The corpses contaminated with spores were also transported away from the plates in similar amounts (*z* = –0.39, *NS*) and at similar rates (Cox regression *coefficient* = 0.09, *z* = 0.5, *NS*) as the controls, and most of them were dragged to the nest. The number of workers that were present around the corpses had no significant effect on the number of hygienic behaviours (GLMM *z* = 0.26, *N* = 156, *NS*) but affected the number of antennations (*z* = 15.56, *P* < 0.001).

**Table 2:** Comparisons of the different fungal treatment types applied on nestmate corpses of *F. polyctena* using fitted mixed-effect models. The *P* values were corrected with the FDR (false discovery rate) method. Significant differences are indicated by bold *P* values.

	Control-treatment			Treatment	
Treatment; *N*	*z*	*P*	Pairwise comparisons	*z*	*P*
**Hygienic behavioural index**					
Spore; 887	2.22	**0.03**	Spore-Hyphae	3.92	** < 0.001**
Hyphae; 887	6.6	** < 0.001**	Spore-Sporulating c.	3.93	** < 0.001**
Sporulating c.; 887	4.87	** < 0.001**	Hyphae-Sporulating c.	0.14	0.885
**Number of antennations**					
Spore; 887	–1.92	0.07	Spore-Hyphae	3.9	** < 0.001**
Hyphae; 887	5.92	** < 0.001**	Spore-Sporulating c.	2.46	**0.02**
Sporulating c.; 887	3.85	** < 0.001**	Hyphae-Sporulating c.	–1.34	0.2
**Removal rate of corpses**					
Spore; 820	1.33	0.19	Spore-Hyphae	3.12	**0.002**
Hyphae; 820	7.94	** < 0.001**	Spore-Sporulating c.	6.59	** < 0.001**
Sporulating c.; 820	5.47	** < 0.001**	Hyphae-Sporulating c.	3.62	** < 0.001**
**Amount of corpses removed**					
Spore; 820	– 0.38	0.79	Spore-Hyphae	2.42	**0.02**
Hyphae; 820	5.00	** < 0.001**	Spore-Sporulating c.	2.34	**0.03**
Sporulating c.; 820	2.52	**0.02**	Hyphae-Sporulating c.	0.11	0.9

Spore—nestmate corpses contaminated with spores; Hyphae—nestmate corpses with hyphae; Sporulating c.—nestmate corpses that are sporulating.

## Discussion

In our study, we showed that by effectively differentiating a high infection risk from a lower one and by avoiding carrying highly infected prey or infected corpses into the nest, ants can use cannibalistic necrophagy as an adaptative feeding strategy to fulfil the nutritional requirements or save the colony from starvation, while still minimising infection risk. A lack of some nutrients or hunger can increase the need for this type of possible food, leading to increased consumption.

We should emphasize here that we did not directly observe consumption of the corpses dragged into the nests. We assumed this based on the weight loss of the paint-marked corpses and the presence of gnawed-out holes in their gasters. However, this finding is consistent with earlier observations on a *F. polyctena* ‘bunker colony’ living in extreme starvation^37^, as well as with those of Mabelis^15^, who also found that the conspecific corpses (victims of the wood ant wars) retrieved into the nest were twice as heavy as those dumped on the waste piles. Moreover, he also proved that the contents of the corpse gasters were consumed and fed to the larvae through regurgitation^6,15^. Field observations also confirm the presence of gnawed-out holes in the gasters of consumed corpses^37^ and suggest that the consumption rate of conspecific corpses is consistent with the current needs of the colony^15,16,37^ – higher in the spring and autumn, when other food sources are scarce.

Based on our results, necrophagy of nestmates in *F. polyctena* seems to be more widespread than previously thought. The workers of *F. polyctena* are able to distinguish among corpses of potential prey, different types of nestmate corpses, conspecific non-nestmates corpses and corpses of ants of alien species. This ability allows them to obtain information from the colony surroundings about potential threats or availability of food sources^43^, thus ensuring the nutrient demand of the colony that can change over time and across situations^44^. Consumption of dead or killed competitors can also be viewed as an efficient means of spreading information among colony members which they had not encountered before, just as cues on food quality and risk during recruitment^45,46^.

Nestmate corpses were brought to the nests at the fastest rate in the starved colonies. In this situation, the largest number of workers gathered around these corpses, which is not surprising because during hunger, most workers begin to forage^11^. The workers from the starved colonies started to consume the corpses already on the plates, but after a while, they transported the corpses to the nest. The corpses brought outside on the cemeteries were almost all considered consumed, indicating that during hunger, the nestmate corpses can serve as an important food source. In hunger condition, a lot of workers die from starvation, so eating their corpses can counterbalance the negative consequences of the stress induced by food deprivation. This result corresponds with other findings in which a food shortage led to a growing rate of conspecific cannibalism in wood ants^15,16^, as well as of both alive individuals and corpses in termites^47,48^ or kin larvae in social bees^12^. On the other hand, conspecific necrophagy in *F. polyctena* also underpins the presence of collective corpse consumption, which enhances the possibility of the spread of diseases^27,28^. These findings suggest that extreme environmental conditions, such as food shortages, may promote corpse necrophagy among relatives in eusocial species. This is also supported by observations of a *F. polyctena* colony trapped in an underground bunker^37^. Moreover, the differences in the reactions of starving and fed colonies led us to the conclusion that the colony reacts in a post-stress situation by using available food sources in larger proportions than usual as if preparing for another similar situation. This is in line with the nature of the species^15,16,37^ and may be associated with the natural response of the colony to changing food availability in nature^44^. Another possibility is that the response is driven by the state-dependent learned valuation, a mechanism that determines the preference for items remembered to be costly or experienced when the subject was in a poor condition^49^. This mechanism, common in vertebrates and already shown also in insects, may help to take quick decisions when neural constraints limit the amount of information processed^49^. However, further studies are needed to clarify the exact nature of this response.

The old corpses of nestmates were transported significantly faster from the plates (mostly directly towards the cemetery) than other kinds of nestmate corpses and those of *C. vagus*. This may be caused by a higher amount of oleic acid on older corpses, which elicit stronger necrophoresis^50–53^. On the other hand, termites, depending on the species, mostly bury or ignore old corpses^52^. Contrary to ants, termites, due to their mode of life, are “trapped” with their dead within their protective nest structures and tunnels^54^ so, it is virtually impossible for them to remove corpses outside. Removing dead relatives may be very important for eusocial insects because sensitivity to specific pathogens among closely related individuals throughout the whole colony can be very similar. In the territorial ant species of the subgenus *Formica* s. str. (wood ants) and the genus *Coptoformica*, dead individuals and insect food remnants are taken out to the border of the territory where they often form spectacular linear ‘cemeteries’^55,56^. However, if not consumed by some subordinate ant species living nearby the territorial colony^57,58^, corpses transported to a cemetery or abandoned somewhere away from the nest might be encountered again, as workers of *F. polyctena* search large areas around their nests. Perhaps ant cannibalism, like in termites, besides nutrient recycling, is also a way to eliminate their dead without the potential risk of re-infections in the future. We observed that corpses from the cemeteries were transported to the nests, probably for consumption. Similar observations were also made under field conditions, when workers dragged conspecific corpses found randomly within their territory (I. Maák & O. Juhász, pers. obs.).

Another possible explanation for the dragging of the corpses into the nest and reducing their weight is destructive disinfection^24^. In pupae, destructive disinfection includes unpacking, grooming, poison spraying and biting, which permanently prevents pathogen multiplication^24^. In the case of corpses, however, this does not seem the case, as most of the nestmate corpses dragged outside were intact, and only rarely were taken into pieces (separate head, thorax and gaster). In addition, only a few (all belonging to *C. vagus*, see Materials and Methods section) of such corpses in pieces were taken into account during the calculation of the consumption rate. Most of the corpses retrieved from the cemetery had only a hole in the gaster, similarly as described by Mabelis^15^ and which was observed in the corpses from the bunker colony^37^.

Our results also showed that the risk of infection caused by corpses might be counterbalanced by effective pathogen recognition and control. The workers of *F. polyctena* reacted in a particular manner according to the given infection threat. During the experiments, the most common response to the fungus-infected corpses was antennation, which also plays a very important role in other social species in the detection of fungal spores on the cuticle or on elements of the habitat^34,59,60^. The detection of fungal parasites may vary between ant species, e.g. with different courses of evolution, life histories and parasite pressures, as well as between different parasites^61^. For example, contrary to *F. polyctena*, the fungus-growing ant *Trachymyrmex* sp. does not change its defensive strategy according to the level of the threat (i.e. the concentration of fungal parasites)^62^. Some ant species can detect infected individuals, but they do not prevent them from entering the nest or discriminate against them in any form^25^. Similarly, wood ants did not show a distinctive behaviour against food contaminated with spores of entomopathogenic fungus that was brought into the nest; however, more self-grooming behaviour was observed in inoculated workers^61^. Moreover, the workers of wood ants, contrary to honeybees^63^, did not increase resin collection in response to brood exposure to *B. bassiana* spores, although a limited effect of resin was found against the germination and growth of this fungus in vitro^64^. On the other hand, the avoidance of environments heavily contaminated with entomopathogenic fungi has been described in other social and non-social insects as well^59,65^.

During auto- and allogrooming, which are the most common hygienic behaviours, antimicrobial secretions from the metapleural and venom glands are spread on the surface of the cuticle, which suppresses the development of fungi and bacteria^19,24,62,66,67^. It seems that formicine ants also create an acidic environment in the stomach by actively ingesting their venom gland secretions, thereby improving individual survival in the face of pathogen contaminated food and limits disease transmission during mutual food exchange (trophallaxis)^68^. This way the infected corpses can elicit only a low-intensity infection in the grooming individuals, inducing the production of specific protective peptides and enzymes that achieve the promptness of the immune system^34,35^. This may be one of the reasons why the workers transported many of the corpses representing lower infection threat (contaminated with spores and hyphae) to the nests, where they were probably consumed. During consumption, these sources of pathogens can actively immunize the workers, which can achieve higher protection and survival^32–34^. These secretions can also be transmitted to other individuals by trophallaxis, improving their protection^69^. Such signals of an infection threat have already been described in other social insects, such as termites, that perform a vibrational display to increase their distance from the non-infected nestmates^70^, and honeybees, in which the cuticular chemical cues of the diseased nestmates can induce an immune response in queens^71^.

In our experiments, the corpses with higher infection levels elicited more intensive responses than those infected only with spores of fungi. Many individuals gathered around such corpses and immediately started to groom them. Similarly, to *F. polyctena*, the workers of the fungus-growing ant *Trachymyrmex* sp. adjust their hygienic behaviours depending on the conidia germination status (germinated or not-germinated) of fungal pathogens^62^. The removal of hyphae is much more difficult than removal of the spores, so sporulating corpses and those infected with hyphae elicited a higher number of hygienic behaviours but also adverse responses, such as biting. Such an elevated number of hygienic behaviours was also observed in other social species, which supports the view that workers can assess the level of risk associated with an infectious agent^6,13,59,62,72–75^. For example, although the foragers of *Mymica rubra* retrieved corpses of prey covered with high number of spores, they consistently avoided sporulating corpses and collected less prey items that had recently died from fungal infection^76^.

Contrary to corpses infected with spores and hyphae, the sporulating corpses were never transported to the nest. Moreover, these corpses were often torn apart or cleaned with formic acid that has strong antifungal and generally antibiotic properties; therefore, it is a very good substance for pathogen control and removal^24,66^. Tearing apart corpses can reduce the size of the suitable environment for the development of the infecting agent and facilitate the treatment of the infected parts with antibacterial secretions^19,24,51,77,78^. This behaviour can be analogous to the already mentioned destructive disinfection of pupae during the incubation period of the fungal pathogen^24^. The developmental stage of the fungal-parasite may be also a key factor in triggering cannibalistic behaviour in termites, as fungus infested moribund ants are cannibalized, however, it seems that once the fungus kills the host, the infected corpse is avoided rather than cannibalized by nestmates^79^. Our observations of *F. polyctena* are consistent with those of Marikovsky (1962), who noted that *Formica rufa* consumed copses infested by a fungus disease, except the sporulating ones, which were never transported into the nest. Transporting corpses to cemeteries can prevent the appearance of at least some of the pathogenic fungi on them, as it was found in the Argentine ant (*Linepithema humile*)^80^. In this species, there are combined toilet and refuse areas, and anal secretions (together with the pygidial gland secretion) of the workers may delay or prevent germination of pathogenic fungi on corpses^80^.

### Conclusions

Although the literature and our findings strongly suggest that cannibalistic necrophagy occurs in social insects, it is essential to search for unambiguous evidence that the corpses are indeed consumed, e.g. by tracking corpse material consumption using fluorescent dusts or dyes^81^. However, our results describing cannibal necrophagy in the ant species suggests that the food recycling hypothesis might be valid in another social insect group besides termites. In this sense, corpses can also be a very efficient means of disease communication, as corpses transported into the nest, combined with trophallaxis, can spread information about a possible threat (e.g. pathogens, rivals, through CHC profiles) and at the same time can also actively immunize the colony. The presence and adaptability of conspecific corpse necrophagy in a certain context should be investigated in several species of different social groups. Such investigations could also clarify the trade-offs between the investment in an efficient pathogen recognition and the efficiency of hygienic behavioural mechanisms. This trade-off can be mediated by several ecological factors (e.g. habitats with different pathogen pressures) and can have many negative and positive consequences regarding species cohabitation or survival. Investment in pathogen recognition and hygienic behavioural mechanisms enable the effective use of an always-available food source, namely, nestmate corpses, increasing the adaptability of the species and the ability to survive even under extreme conditions.

## Methods

### Study species and colonies

*Formica polyctena* Förster, 1850 is an oligotope of temperate and sub-mountain coniferous, mixed or deciduous forests, usually occurring at the edge of the forest and along intra-forest roads, but often also deep inside the forest. Nests are large and built from fine plant material^38,39^. Colonies are usually highly polygynous with up to few thousand queens and over a million of workers. It often forms multi-nest (polydomous) systems, and its foraging trails cover a large extent of the forest floor^39^. As a competitively strong, top dominant species, it exerts a high pressure on other ant species inside its territory, and reduces the density of many invertebrates. Besides collecting honeydew in all layers of vegetation from roots to the crown of trees, it also scavenges and predates on small animals occurring on the forest floor and in the canopy^38,39^.

The 20 *F. polyctena* sub-colonies were collected from a planted Scots pine (*Pinus sylvestris*) forest near the village of Ásotthalom (southern Hungary) from colonies situated at least 250 m from each other. Workers from a *Camponotus vagus* colony were also collected in the aforementioned location, the corpses of which were used further in the experiment as allospecific control. We used this species because the corpses of major *C. vagus* workers can serve as an important food source for an ‘always hungry’ *F. polyctena* colony^82^.

Laboratory colonies (i.e. sub-colonies taken from the field) were set up, each containing ca 2000 workers, some brood, and original nest material. The colonies were kept under constant laboratory conditions (temperature 23 ± 2 °C; 42–43% RH; 12L:12D cycle) in plastic boxes (44 cm × 31 cm × 23 cm) with their cover cut in a circular part (Ø 15 cm) and covered with a fine wired metal mesh for proper ventilation and an easier moistening. Each box containing a colony was connected with a 10 cm long plastic tube to a foraging arena (60 cm × 30 cm × 15 cm). They were watered daily and fed every second day with a commonly used artificial diet^83^ in a distant part of each foraging arena.

### Laboratory Experiments

In each experimental setup, we used 10 freeze-killed corpses (per colony) of healthy and uninjured individuals of different types and presented them to the experimental colonies. Corpses were obtained by placing individuals in the freezer (< –18 °C) to avoid altering their cuticular hydrocarbon (CHC) profile^84^. The corpses were placed 10 cm from the foraging arena entrance on a plastic plate (Ø 9 cm) and observed for one minute. The observations were repeated every three minutes for one hour. During the observations, we noted (1) the number of corpses on the plates at the beginning and at the end of each 1-min observation, (2) the number of workers on the plate, and (3) their behavioural responses to the corpses. The responses were the following: ignoring, antennation, mandible spreading, biting, dragging and retreating in the corpse consumption experiment as well as grooming and spraying with formic acid in the fungus infection experiment^43,74^. When a corpse was carried away, we also recorded the direction of movement, i.e. to the nest or to the cemetery. We defined cemeteries as large piles of corpses and other wastes. They were made by each of our studied colony outside in the foraging arena. Plates were cleaned after every experimental setup.

#### Behavioural responses to the corpses of potential prey and nestmates

Before the corpse consumption experiments, we performed additional experiments with corpses of a potential prey species, *Drosophila melanogaster* using four colonies of *F. polyctena.* The behaviour elicited by these corpses was compared with the responses to the corpses of nestmates and non-nestmates.

#### Nestmate corpse consumption and the role of starvation

Six colonies (four used also in the experiment presented before) of *F. polyctena* and one colony of *C. vagus* were used for these experiments*.* The nestmate corpses taken from these colonies were used during the experiments after being paint-marked with an individual colour combination separately on their head, mesosoma and gaster with Art Deco enamel paints of different colours. After painting, the weight of each corpse was measured with an OHaus Explorer Pro EP 214 precision analytical balance (SD ± 0.1 mg).

The first corpse consumption experiment was performed with corpses of *C. vagus* (*N* = 60 corpses). This was followed by the control experiment with nestmate corpses (NC; *N* = 60). After this, the colonies were subjected to a seven-day starvation period, and the treatment with NC was repeated (starved colonies; *N* = 60). After inducing feeding stress, we again applied the normal feeding protocol for a week and then repeated the NC treatment once more (non-starved colonies; *N* = 60) to check if there was a compensatory response of *F. polyctena* workers after the feeding stress. This process was followed by the experiment with seven-day-old nestmate corpses (old NC; *N* = 60). Each corpse type was treated as described previously. As the aim of this study was to assess the state dependent response of colonies, we were not able to randomize between the setups, so to fulfil the experimental requirements and to avoid the influence of the experimental order, we left a week time gap between the different experiments.

In each experimental setup, we put a number of individually marked corpses (obtained in the same manner as the experimental ones) to an empty arena to control for the weight loss during ageing and desiccation as follows: 17 corpses of *C. vagus*, 13 of NC, 28 of NC in starved colonies, and 29 of NC in non-starved colonies. The only exception was the old NC setup, where we performed the experiment without controls. In this case, the corpses were already seven days old, so the further weight loss due to desiccation were barely detectable. Following each setup of the corpse consumption experiment, we checked regularly (on a daily basis at the time of the experiment) for five days the arenas for marked corpses/or corpse parts brought from the nest to the cemetery. If corpses or corpse parts appeared, they were measured with the analytical balance. In each case, the control corpses were also measured for the comparison of weight loss differences. Control corpses damaged during handling were excluded from further measurements.

To determine whether a corpse was consumed, we measured the weight loss of corpses by subtracting the weight of corpses before the experiment and after they were brought out from the nest (*W*_*lost*_ = *W*_*before exp* –_ *W*_*after out*_). If the difference was larger than two times the mean weight loss measured during the desiccation, the corpses were considered consumed. The consumption of corpses was also visually confirmed by noticing a gnawed-out hole on the gaster or other parts of the body. Only those corpses that were brought out in one piece were considered (*N*_*cons*_ = 78, *N*_*n-cons*_ = 78). In addition, in two cases (both in *C. vagus*) all corpse parts (head, mesosoma, gaster) were brought out on the same day, so these were also considered. Otherwise, it was impossible to carry out the comparison with the controls, which were whole corpses. However, we are aware that these data are only circumstantial evidence of the corpse consumption, as it may also indicate the processing of the corpses without consumption. Moreover, gnawed holes and corpse fragmentation can accelerate desiccation, which could simulate consumption.

#### Pathogen recognition

These experiments were performed firstly with eight colonies and then repeated with six colonies of *F. polyctena*. We decided to repeat the experiments on separate colonies because of the possible effect of previous experience. We infected the corpses with the entomopathogenic fungus *Beauveria bassiana* SZMC 12656, a widely used species for laboratory studies^85,86^.

The fungus was maintained on Malt Extract Agar (MEA) (1% glucose, 0.5% yeast extract, 0.5% malt extract, 2% agar) at room temperature. For the experiments, conidia were washed from 14 days old cultures with distilled water. Spore number was assessed with Bürker-chamber.

After sterilizing the freeze-killed corpses with UV irradiation for 30 min, they were laid on sterile, wet cotton wool on 24-well plates. Infection was maintained by dropping 3 μl of 10^8^ conidia/ml inoculum on the head and gaster of the corpses. We took great care to apply the conidia evenly of the surface of the corpses in such a way that the spores remained (at least the great majority) on the corpses. For analysing reactions for only conidia, the inoculum was let to dry, and the corpses were used 10 min after infection. For the other experiments, plates were incubated in darkness at 25 °C for five (only hyphae) or eight days (hyphae with conidia). The different development stages of the fungus were identified under a stereoscopic microscope^85^.

Different developmental stages of the fungi were used: conidia (referred to as spore-contaminated), hyphae (referred to as with hyphae) and hyphae with conidia (referred to as sprorulating corpses). In the experiments with fungal conidia (sporulating corpses), only seven (first set of experiments) and five colonies (repeated experiments) were used because of the contamination of their corpses with other types of fungi. In each infection stage (3 categories), 10 untreated corpses per colony were used as a control, handled like the infected ones. Here, data collection was the same as in the experiments described above (see Laboratory experiments). We should note, however, that the method of producing different pathogen levels used in this study does not fully mimic real pathogen progression^87^. On the other hand, this entomopathogenic fungus occurs at high densities in the soil and on corpses^40^, and can be responsible for natural infections of ants in field populations^41^. Presumably, such encounters of wood ant workers with infected (nestmate) corpses in different stages can frequently happen in nature as well.

### Statistical analysis

#### Responses to the corpses of potential prey and nestmates

Besides the comparison of responses to the corpses of potential prey, nestmates and non-nestmates, we also compared the behavioural responses to nestmate corpses (2 h control) used during the different setups (altogether 18 colonies) to test for reaction consistency.

Generalized Linear Mixed Models (GLMM, binomial error, maximum likelihood fit) were used to analyse the effect of corpse type and other variables on the behavioural responses recorded in the course of each 1-min observation. An aggression index was calculated for each 1-min observation where the number of adverse responses was divided by the total number of behavioural responses. Mandible spreading, biting, dragging, and retreat were considered as adverse responses^43^. In the full model, the origin of corpses was included as a fixed factor, the number of ant workers at the end of each 1-min observation as a covariate, and the observation period and nest ID as random factors.

Similar generalized linear mixed models (GLMM, negative binomial error, maximum likelihood fit) were used to test the effect of corpse type and the number of corpses on the number of workers recorded in the course of each 1-min observation. Data were considered only from those 1-min observations where there were still corpses on the plate (*N* = 180 for prey comparison; *N* = 248 for the nestmate comparison).

The corpse removal rate from the plates was analysed with the Cox regression model (proportional hazard approach, *N*_*prey*_= 120; *N*_*nestmate*_= 180 corpses). The origin of corpses was included as a dummy variable, while nest ID was included as a random factor in order to handle dependencies. The effect of corpse type on the decision whether or not to remove corpses from the plate was analysed using GLMM approach (binomial error, maximum likelihood fit; *N*_*removed*_= 70, *N*_*n-removed*_ = 50 for prey comparison, *N*_*removed*_= 150, *N*_*n-removed*_ = 30 for nestmate comparison). In the full model, corpse type was introduced as a fixed factor and nest ID as a random factor.

#### Nestmate corpse consumption and the role of starvation

The corpse removal rate from the plates (*N* = 10 corpses per colony in 5 different setups; *N*_*total*_ = 300 corpses) and the rate of external corpse disposal during the five consecutive days (proportional hazard approach, *N*_*recovered*_= 256, *N*_*n-recovered*_ = 44) were tested with Cox regression models (proportional hazard approach). The origin of corpses was included as a dummy variable, while nest ID was included as a random factor to handle dependencies. The decision to remove a corpse from the plates (*N*_*removed*_= 266, *N*_*n-removed*_ = 34) and to carry a corpse inside the nest or leave it in the arena (GLMM, binomial error, maximum likelihood fit, *N*_*intonest*_ = 226, *N*_*outside*_ = 74) and the number of ant workers present on the plates (GLMM, negative binomial error, maximum likelihood fit) were also analysed with GLMM model constructions (see previous section). In these analyses, all corpses were included (*N* = 300).

The effect of corpse type on whether the corpses brought out were chopped into pieces (*N* = 96) or left in one piece (*N* = 160) and whether they were consumed was analysed with the GLMM approach (binomial error, maximum likelihood fit; *N* = 156), where corpse type was introduced in the models as a fixed factor and nest ID as a random factor.

#### Pathogen recognition

The effect of corpse type and other variables on the hygienic behavioural responses recorded in the course of each 1-min observation was analysed using generalized linear mixed models (GLMM, binomial error, maximum likelihood fit). An index was calculated for each 1-min observation where the number of hygienic responses was divided by the total number of behavioural responses. Grooming, dragging and spraying with formic acid were considered hygienic responses^43^. In the full model, the origin of corpses was included as a fixed factor, the number of ant workers at the end of each 1-min observation as a covariate, and nest ID as a random factor. Similar generalized linear mixed models (GLMM, negative binomial error, maximum likelihood fit) were used to test the effect of corpse type and the number of workers that appeared around the corpses on the number of antennations recorded in the course of each 1-min observation. Data only from those 1-min observations where there were still corpses on the plate were considered (*N* = 887).

The corpse removal rate from the plates was tested with Cox regression model (proportional hazard approach, *N* = 820). The origin of corpses was included as a dummy variable, while nest ID was included as a random factor to handle dependencies. The effect of corpse type on the decision whether or not to remove corpses from the plate was analysed using GLMM approach (GLMM, binomial error, maximum likelihood fit, *N*_*removed*_= 712, *N*_*n-removed*_ = 108). In the full model, corpse type was introduced as a fixed factor and nest ID as a random factor. Here, all the corpses were included (*N* = 820).

All statistical analyses were carried out in the R Statistical Environment^88^. Cox regression analysis was carried out with the use of *coxme* function in *coxme* package^89^. GLMMs were performed using *glmer* function in *lme4* package^90^, automated model selection with the help of *dredge* function in *MuMIn* package^91^. The effects of different explanatory factors and variables were averaged across the best models with delta < 2^92^. *Relevel* function was used in order to carry out post-hoc sequential comparisons among factor levels when performing GLMM and Cox regression analyses. We applied table-wide sequential FDR (False Discovery Rate) correction to reveal the exact significance levels in these cases.

## Data Availability

The datasets generated and/or analysed during the current study are available from the corresponding author on reasonable request.
